# Progressive Multifocal Leukoencephalopathy Associated With Idiopathic CD8+ Lymphocytopenia

**DOI:** 10.7759/cureus.32870

**Published:** 2022-12-23

**Authors:** Marta Lopes, Ana Monteiro, Maria do Céu Dória, André Rêgo, Marta Rocha, Daniela Madeira, Teresa Valido

**Affiliations:** 1 Internal Medicine, Hospital Professor Doutor Fernando Fonseca, Amadora, PRT; 2 Neurology, Hospital Professor Doutor Fernando Fonseca, Amadora, PRT

**Keywords:** hiv-negative progressive multifocal leukoencephalopathy, idiopathic cd8+ lymphocytopenia, pembrolizumab, john cunningham virus, progressive multifocal leukoencephalopathy

## Abstract

Progressive multifocal leukoencephalopathy (PML) is a severe demyelinating disease of the central nervous system (CNS) caused by the polyoma John Cunningham (JC) virus. This virus is rarely pathogenic in immunocompetent individuals, being associated with profound cellular immunosuppression.

We present a case of a 72-year-old woman with schizoaffective disorder who presented to the emergency department with dysarthria and right hemiataxia. The initial computer tomography was normal and the diagnosis of ischemic stroke was first assumed. However, during hospitalization there was a progressive worsening of symptoms with cerebellar ataxia, and the magnetic resonance revealed a lesion in the right middle cerebellar peduncle hypointense in T1 and hyperintense on T2/fluid attenuated inversion recovery (FLAIR) sequence, suggestive of PML. Although the first cerebrospinal fluid analysis was negative, the second one was positive for the JC virus. Furthermore, due to radiological and clinical progression, mirtazapine was started and the patient underwent a course of intravenous immunoglobulin, with no response. In parallel, causes of immunosuppression were investigated, which led to the diagnosis of idiopathic CD8+ lymphocytopenia. Due to rapid progression of symptoms and radiological worsening of lesions, pembrolizumab was administered. After the first administration of pembrolizumab there was a transitory clinical stabilization. However, shortly after the second administration of pembrolizumab, the patient developed stridor with bilateral vocal cord paralysis and subsequent symptom progression, which led to the death of the patient three months after the appearance of initial symptoms.

In conclusion, we report a case of a PML in a patient with idiopathic CD8+ lymphocytopenia, enhancing the need for a high suspicion index for this entity as well as for occult and less frequent forms of immunosuppression. Although there have been various case reports of favourable outcomes with pembrolizumab for PML, more research is needed, particularly to identify patient factors that might be associated with better responses to this therapy.

## Introduction

Progressive multifocal leukoencephalopathy (PML) is a severe demyelinating disease of the central nervous system (CNS) caused by the polyoma John Cunningham (JC) virus. This virus is rarely pathogenic in immunocompetent individuals, being associated with profound cellular immunosuppression, which allows replication-driven neurotropic JC virus to cause lytic infection of oligodendrocytes and glial cells, leading to progressive neurological decline unless immune reconstitution occurs [1].

The human immunodeficiency virus (HIV) infection has been the most frequent underlying predisposing factor of PML, occurring in more than one-half of individuals, however, there has been a significant decline since the advent of highly active antiretroviral therapy (HAART). Moreover, there has been a rise in this condition in transplant patients as well as patients under immunosuppressive drugs, particularly natalizumab and efalizumab. While some case reports have documented PML in individuals with minimal or occult immunosuppression, such cases are very rare and their pathogenesis is not well understood, as in this case [2-4].

There are no effective antiviral therapies for JC virus available to date. In individuals in whom the immune dysfunction can be restored, either through withdrawal of immunosuppressive drugs or HAART in HIV patients, survival is improved. In contrast, PML in the context of hematologic malignancy or primary immunodeficiency is lethal in the majority of cases as there are no therapeutic options for immune reconstitution [2,4].

Pembrolizumab is a monoclonal antibody that acts as a checkpoint inhibitor disrupting interactions of programmed cell death protein 1 (PD-1) on T cells and its ligands on antigen-presenting cells. The binding of programmed cell death ligand 1 (PDL-1) to its programmed cell death 1 (PD-1) receptor on activated T-cells leads to the inhibition of cytotoxic T-cell response. It is hypothesized that in PML patients PD-1 expression is increased in cerebrospinal fluid (CSF) CD4+ and CD8+ lymphocytes. Also, there is laboratory evidence of PD-1 upregulation on T cells in PML patients, which led to the off-label use of this drug in PML patients where no other option was available. Although there are not to date any randomized controlled trials for this therapy in PML, there have been many case reports some of which had favourable outcomes ranging from stabilization to moderate improvement of symptoms [5-7].

## Case presentation

We present a case of a 72-year-old woman who was born in Cape Verde and had been living in Portugal for over two decades. She had schizoaffective disorder and was medicated with oral risperidone and monthly depot injections of haloperidol decanoate.

She presented to the emergency department due to a fall from her own height in her residence, after which her son noted dysarthria and right lower limb weakness when he went to visit her later that day. She was unable to ascertain the time of installation of the symptoms. On hospital admission, neurological examination revealed moderate dysarthria and right hemiataxia. The cranial computed tomography (CT) was normal. At this point, an ischemic stroke of the right hemisphere with unknown time of symptom onset was suspected. Hence, she was started on acetylsalicylic acid as well as statin and was admitted for etiological investigation. The transthoracic echocardiogram was normal, the carotid ultrasound did not reveal any plaques with haemodynamic significance and the 24-hour Holter monitor did not detect any arrhythmias. Furthermore, she was not a smoker and other cerebrovascular risk factors, namely hypertension, dyslipidaemia and diabetes mellitus, were excluded. Initial laboratory findings were unremarkable except for vitamin B12 deficiency, for which she initiated parenteral supplementation.

**Figure 1:**
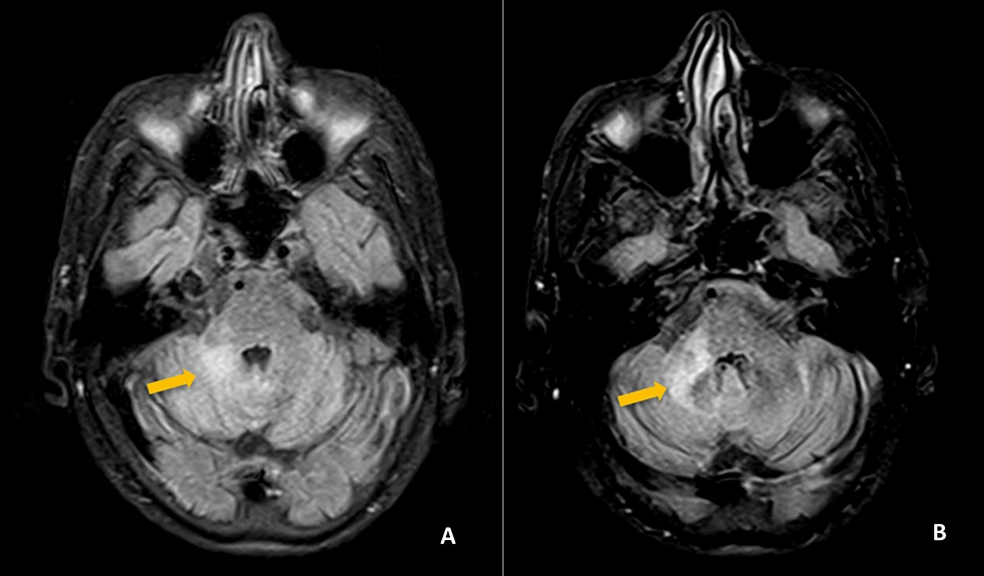
First brain MRI two weeks after symptom initiation, transverse T2/FLAIR sequence (A and B) with hyperintense lesion on the right cerebellar peduncle (yellow arrows). FLAIR: fluid attenuated inversion recovery

During the first week after admission, there was a progressive worsening of the dysarthria, accompanied by right hemiataxia, incoercible vomiting and vertigo. She repeated the cranial CT-scan which did not reveal any new lesions. The magnetic resonance (MRI) performed two weeks after admission revealed a lesion in the right middle cerebellar peduncle hypointense in T1 and hyperintense signal on T2/fluid attenuated inversion recovery (FLAIR) sequence, without diffusion restriction or edema and without enhancement after gadolinium administration, suggestive of PML (Figure 1). The CSF analysis revealed 2 cel/uL, a slightly elevated protein count (46 mg/dL) and normal glucose levels (78 mg/dL in CSF and 90 mg/dL in serum), without presence of oligoclonal bands. The CSF polymerase chain reaction (PCR) for herpes virus and the JC virus was negative and the microbiological exam of the CSF was also negative. The autoimmune in serum and CSF panels were negative. The anti-myelin oligodendrocyte glycoprotein and anti-aquaporin 4 antibodies were also negative.

**Figure 2:**
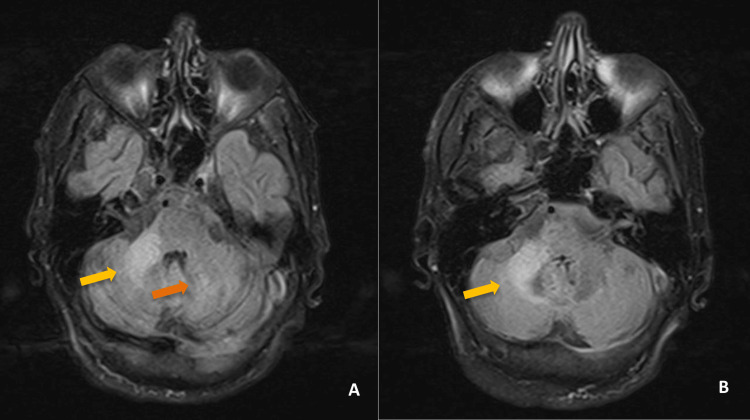
Second brain MRI 1 month after the previous one, transverse T2/FLAIR sequence (A and B) with increased size of the peduncular lesion (yellow arrows) and appearance of a new lesion on the contralateral cerebellar hemisphere with the same characteristics (orange arrow). FLAIR: fluid attenuated inversion recovery

**Figure 3:**
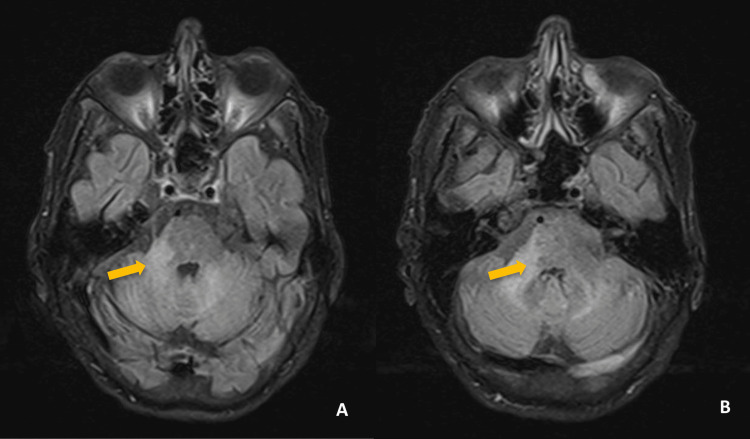
Third brain MRI performed three weeks after the previous one, transverse T2/FLAIR sequence (A and B), with a new lesion on the right brainstem (yellow arrows). FLAIR: fluid attenuated inversion recovery

Due to progression of the cerebellar symptoms and radiological worsening on the MRI performed one month after the initial one, with increased size of the peduncular lesion and appearance of a new lesion on the contralateral cerebellar hemisphere with the same characteristics (Figure 2), a course of intravenous immunoglobulin (IVIG) was proposed assuming the probable diagnosis of PML. The CSF analysis was repeated before initiation of IVIG and was positive for the JC virus (PCR JC virus 964 copies/ml), therefore confirming the radiological and clinical hypothesis. The patient underwent a course of five days of IVIG (0.4 g/kg/day) and was also started on mirtazapine 15mg with no clinical improvement. Afterwards, rapid symptom progression ensued with the development of bilateral facial paralysis, right abducent nerve paralysis as well as bulbar symptoms, namely dysphonia and dysphagia, with the appearance of a new lesion in the right brainstem on MRI performed three weeks after the previous one (Figure 3).

Therefore, after multidisciplinary discussion and bibliographic consultation, it was decided to administer pembrolizumab (2mg/kg every four weeks).

In parallel, causes of immunosuppression were investigated. The serologies for HIV and hepatitis B and C were negative. The patient never underwent any immunosuppressive medication. Although the hemogram did not reveal any cytopenia, further testing revealed an isolated CD8+ lymphocytopenia of 69 cel/uL (5% of the total lymphocyte count) with normal CD4+, natural killer and B lymphocyte counts. Furthermore, paraneoplastic and autoimmune causes of immunosuppression as well as hypogammaglobulinemia and secondary CD8+ lymphocytopenia were also excluded.

After the first administration of pembrolizumab there was a transitory stabilization of symptom progression with dysphagia improvement. However, two days after the second administration of pembrolizumab, the patient developed stridor with bilateral vocal cord paralysis. An emergent tracheostomy was performed, however, due to nosocomial pneumonia the patient died, approximately three months after the appearance of the initial symptoms.

## Discussion

PML occurs almost exclusively in patients with immunosuppression. The presentation of PML is usually subacute and manifests as progressive, multifocal neurological deficits that vary depending on the site of the lesion and therefore comprises a wide spectrum of symptoms. Areas commonly involved include the subcortical white matter, periventricular areas, and cerebellar peduncles, usually sparing the optic nerve and the spinal cord. On MRI, they appear as hypointense areas on T1-weighted images and hyperintense lesions on T2-weighted sequences [7]. Although there was a need for tissue biopsy in the past, nowadays the diagnosis is mainly based on clinical and radiological features as well as the demonstration of the JC virus in the CSF which has a high sensitivity and specificity [8].

In our patient, the absence of and evident cause of immunosuppression and seemingly acute installation of the initial symptoms, led to the initial hypothesis of a stroke, which was later abandoned as symptoms progressed. Furthermore, there was also a rapid disease progression throughout a three-month period with a clear clustering of lesions on the cerebellar peduncles and hemispheres, later progressing to the brainstem with invasion of the nucleus ambiguous causing bilateral vocal cord paralysis. Although the imaging was typical of PML, the initial CSF was able to exclude multiple infections as well as inflammatory causes, but was negative for the JC virus. In some cases, PML can occur with negative PCR for the JC virus in CSF, which does not exclude the diagnosis. The second CSF analysis confirmed the presence of the virus, which together with the clinical and radiological findings confirmed the diagnosis obviating the need for a CNS biopsy.

Our case is also particular since there was an idiopathic CD8+ lymphocytopenia, an underlying rare cause of immunosuppression, for which there were no treatment options available for immune restoration. Isolated CD8+ T-lymphocyte deficiency is a rare entity and its clinical implications are poorly understood. JCV-specific CD8+ T-lymphocytes actively destroy JC virus-infected glial cells, thereby containing PML. This disease has been linked to an isolated CD8+ T-lymphocytopenia in one case report, and this association could be related to the role of cytotoxic T-lymphocytes in containing the infection [9].

As far as treatment options were concerned, given the rapid progression of symptoms treatment with IVIG was first attempted before diagnosis confirmation. IVIG has been postulated to generate an immune response against JC virus and has therefore been used in patients with PML and use of immunosuppressant drugs such as natalizumab [10].

Furthermore, since in vitro studies have also shown that JC virus infection of oligodendrocytes is serotonin 5HT2A receptor-dependent, this led to the postulation that 5HT2A antagonists like mirtazapine might be beneficial in the treatment of PML by preventing the spread of the virus. Although this agent has been empirically used as adjunctive therapy in PML patients, there has not been definite evidence of this therapy [11].

Both these therapeutic attempts were unsuccessful, with sustained neurological symptom progression which led to the decision to use pembrolizumab. Due to its mechanisms of action, this monoclonal antibody has been used recently in PML patients, especially in those where no other options for immune restoration were available, which was the case of our patient. Besides having some favourable clinical outcomes, some case reports have shown a reduction in the JC viral load in the CSF and an increase in in vitro CD4+ and CD8+ anti-JC virus activity [6]. Despite this, there have also been cases of symptom worsening after pembrolizumab, which was the case with our patient.

## Conclusions

As far as the PML infection is concerned, regardless of the underlying immunosuppressive factor, one- and five-year survival rates have been estimated to be 30% and 10%, respectively. The chances of survival depend mainly on whether immune function can be restored.

We report a case of a PML in a patient with idiopathic CD8+ lymphocytopenia, enhancing the need for a high suspicion index for this entity as well as for occult and less frequent forms of immunosuppression. Although there have been various case reports of favourable outcomes with pembrolizumab for PML, more research is needed, particularly to identify patient factors that might be associated with better responses to this drug.
